# Promoter hypomethylation drives *ABCB1*-mediated carfilzomib resistance in multiple myeloma

**DOI:** 10.1186/s13148-026-02115-y

**Published:** 2026-04-01

**Authors:** Seungbin Han, Larissa Haertle, Umair Munawar, Marietta Truger, Ann-Sophie Hainold, Chien-Yun Lee, Shilpa Kurian, Christina Verbruggen, Silvia Nerreter, Cornelia Vogt, Emma Besant, Nina Rein, Johanna Lehmann, Max Köppel, Yoko Tamamushi, Xiang Zhou, Torsten Steinbrunn, Thomas Haaf, Bernhard Küster, Ondrej Slaby, Claudia Haferlach, Hermann Einsele, Leo Rasche, Johannes Waldschmidt, Andrej Besse, Christoph Driessen, Lenka Besse, K. Martin Kortüm

**Affiliations:** 1 Department of Internal Medicine II, Department of Hematology and Oncology, University Hospital of Wuerzburg, Oberduerrbacher Str.6, 97080 Wuerzburg, Germany; 2 TranslaTUM, Center for Translational Cancer Research, Technical University of Munich, Munich, Germany; 3 Munich Leukemia Laboratory (MLL), Munich, Germany; 4 School of Life Sciences, Technical University of Munich, Munich, Germany; 5 Department of Medical Oncology, Dana-Farber Cancer Institute, Harvard Medical School, Boston, MA USA; 6 Institute of Human Genetics, Julius Maximilians University Wuerzburg, Wuerzburg, Germany; 7 Department of Biology, Masaryk University, Brno, Czech Republic; 8 Laboratory of Experimental Oncology, Cantonal Hospital St. Gallen, St. Gallen, Switzerland

**Keywords:** Multiple myeloma, Multi-omics, Epigenetic regulation, Proteasome inhibitor resistance, ABCB1, DNA methylation

## Abstract

**Background:**

Proteasome inhibitors (PIs) are indispensable for the treatment of multiple myeloma (MM), the second most common hematologic malignancy. Although primary resistance to PIs is rare, most patients eventually relapse and develop acquired resistance, with underlying mechanisms that remain incompletely understood and appear to be drug-specific. In the case of bortezomib, resistance is often associated with *PSMB5* mutations. In contrast, resistance to carfilzomib (CFZ) is mediated by overexpression of the drug efflux transporter ABCB1. However, the regulatory mechanisms driving ABCB1 upregulation in CFZ-resistant MM remain unclear.

**Methods:**

An integrative multi-omics analysis was conducted using paired samples from a CFZ-sensitive and -resistant MM patient, alongside resistant cell line models. Whole-genome sequencing (WGS), whole-genome bisulfite sequencing (WGBS), and RNA sequencing (RNA-seq) were used to assess the genotype (structural variants, single nucleotide variants, and copy number variations), methylation status, and the expression of the *ABCB1* locus. *ABCB1* promoter methylation levels and expression levels in an independent MM subcohort were analyzed to determine clinical relevance. Functional validation was performed using dual-luciferase reporter assays, *DNMT1* knockdown, and treatment with DNA methyltransferase inhibitors (DNMTis) to evaluate methylation-dependent regulation of *ABCB1* expression.

**Results:**

Significant hypomethylation of the *ABCB1* downstream promoter region was identified (GH07J087598) in a CFZ-resistant patient sample, which correlated with elevated *ABCB1* expression. Consistent with the paired CFZ-resistant case, the independent MM subcohort showed a significant inverse association between *ABCB1* promoter methylation and *ABCB1* expression. These findings align with results obtained from CFZ-resistant MM cell line models, which demonstrated reduced promoter methylation and elevated *ABCB1* expression compared to their wild-type counterparts. Furthermore, treatment with DNA methyltransferase inhibitors as well as *DNMT1* knockdown enhanced *ABCB1* expression while demethylating the promoter, thereby validating the functional significance of promoter hypomethylation in *ABCB1* overexpression.

**Conclusions:**

Our findings highlight *ABCB1* promoter hypomethylation as a potential epigenetic driver of CFZ resistance in MM. These results underscore the clinical relevance of epigenetic regulation in drug resistance and the potential of targeting DNA methylation as a therapeutic strategy to overcome resistance in MM.

**Supplementary Information:**

The online version contains supplementary material available at 10.1186/s13148-026-02115-y.

## Introduction

Multiple myeloma (MM) is a clonal plasma cell malignancy and remains the second most common hematologic cancer [1, 2]. Proteasome inhibitors (PIs), including bortezomib (BTZ), carfilzomib (CFZ), and ixazomib, are widely used in both newly diagnosed and relapsed/refractory MM [3, 4]. Their primary mechanism of action involves the inhibition of the ubiquitin–proteasome system, leading to the accumulation of misfolded proteins, elevated cellular stress, and apoptosis [5, 6]. Owing to their proven clinical efficacy, virtually all patients with MM are exposed to PIs as part of combination therapies; however, among those who can tolerate therapy for longer timeframes, the vast majority eventually develops drug resistance [7–9]. Known mechanisms of tumor-intrinsic resistance to proteasome inhibitors involve both genetic and epigenetic alterations [9, 10]. Among genetic events, mutations in the proteasome subunit *PSMB5* are the most extensively studied. These typically occur within the β5 subunit’s substrate-binding pocket and impair the interaction between PIs and the chymotrypsin-like active site, thereby reducing inhibitor affinity and proteolytic inhibition [11, 12]. In parallel, epigenetic remodeling has also been proposed as a possible resistance mechanism, mostly through altered promoter methylation of *PSMD5*, which encodes a regulatory chaperone essential for 26S proteasome assembly. Hypermethylation of the *PSMD5* promoter has been observed in a subset of PI-refractory patients and is associated with reduced gene expression, elevated proteasome biogenesis, and diminished PI sensitivity [13].

Beyond proteasomal adaptations, increased expression of *ABCB1*, a member of the ATP-binding cassette transporter family, plays a prominent role in PI resistance [14]. *ABCB1* encodes a transmembrane efflux pump that actively exports a broad range of xenobiotics, including CFZ, thereby reducing intracellular drug accumulation and attenuating therapeutic efficacy [15, 16]. Although *ABCB1* overexpression is frequently observed in PI-resistant MM, the upstream regulatory mechanisms governing its transcription remain unclear. In other malignancies, promoter hypomethylation has been implicated in *ABCB1* activation and has been linked to resistance against various chemotherapeutic agents [17, 18]. Whether such epigenetic regulation contributes to *ABCB1*-mediated PI resistance in MM has yet to be explored.

In this study, we aimed to determine whether DNA methylation contributes to *ABCB1* regulation in the context of proteasome inhibitor resistance in MM. We performed genomic, epigenomic, and transcriptomic profiling of paired samples from a patient before and after developing CFZ resistance, and validated our findings in matched PI-sensitive and -resistant cell line models. Our multi-omics approach included whole-genome sequencing (WGS), whole-genome bisulfite sequencing (WGBS) and RNA sequencing (RNA-seq) to assess potential epigenetic alterations. To assess clinical relevance, we further examined *ABCB1* promoter methylation and *ABCB1* expression in an independent MM subcohort (*n* = 13). Finally, functional validation was performed using a CpG-free dual-luciferase reporter assay [19], *DNMT1* knockdown (KD), and DNA methyltransferase inhibitor (DNMTi) treatment.

## Methods

### Patient samples

Sequential primary samples were obtained from peripheral blood of a patient with an initial diagnosis of MM who progressed to plasma cell leukemia, during routine diagnostic procedures at the Cantonal Hospital St. Gallen (Switzerland). Sample 1 was taken at relapse after a BTZ-containing regimen; sample 2 was obtained upon progression on a pomalidomide and CFZ combination therapy. In addition, bone marrow aspirates were obtained from 13 patients with MM, who were treated at the University Hospital of Wuerzburg (Germany). CD138^+^ plasma cells were isolated using magnetic-activated cell sorting with anti-CD138 microbeads (Miltenyi Biotec, Bergisch Gladbach, Germany) according to the manufacturer’s protocol.

### Cell culture

Human MM cell lines (AMO1, ARH77, L363; obtained from the Leibniz Institute DSMZ, Braunschweig, Germany) were cultured in RPMI-1640 medium supplemented with 10% fetal bovine serum, sodium pyruvate, L-glutamine, and penicillin/streptomycin. CFZ-resistant derivatives (AMO1CFZ, ARH77CFZ, L363CFZ) were established by gradually exposing parental cells to increasing concentrations of CFZ over several months, as previously described [20], and subsequently maintained in 90 nM CFZ. The cells were routinely tested for mycoplasma contamination and short tandem repeat (STR)-typed (DSMZ) to confirm authenticity.

### DNA methyltransferase inhibitor treatment

For DNA methyltransferase inhibition, CFZ-resistant ARH77 cells and the wild-type (WT) were treated with either 25 nM decitabine (Dec) or 1 µM GSK-3484862 (GSK) (MedChemExpress, Monmouth Junction, USA) for 7 days, with media replenished every 48–72 h. On day 7, 1 × 10^6^ cells were harvested for analysis of *ABCB1* expression and promoter methylation.

### DNMT1 siRNA knockdown

ARH77 cells (2 × 10^6^ per reaction) were washed with PBS, resuspended in NeonNxt™ Resuspension R Buffer (Thermo Fisher Scientific GmbH, Dreieich, Germany), and mixed with siRNA targeting DNMT1 (Thermo Fisher Scientific, #4390824) or a non-targeting control (Thermo Fisher Scientific, #4390843) to a final concentration of 2 µM. Electroporation was performed with the NeonNxt™ Transfection System at 1450 V, 10 ms, 3 pulses. Immediately after pulsing, cells were transferred to pre-warmed, antibiotic-free RPMI-1640 complete medium in 6-well plates and incubated at 37 °C with 5% CO_2_. The downstream effects of DNMT1 KD were assessed at days 3, 5, and 7 post-transfection. *DNMT1* and *ABCB1* expression were quantified by RT-qPCR and CFZ sensitivity was measured using the alamarBlue assay.

### CFZ sensitivity

CFZ sensitivity was assessed using the alamarBlue assay. Cells (1.8 × 10^4^ per well) were seeded in 96-well flat-bottom plates in RPMI-1640 complete medium and treated for 72 h with CFZ (MedChemExpress, Monmouth Junction, USA) at the indicated concentrations. After 72 h, alamarBlue reagent was added at 10% of the well volume and the absorbance was measured at 570 and 600 nm using a Tecan Infinite 200 Pro plate reader (Tecan Deutschland GmbH, Crailsheim, Germany). IC_50_ values were calculated by nonlinear regression in GraphPad Prism software (v.10.1.2).

### Multi-omics analysis

Whole-genome sequencing (WGS) libraries were prepared using the TruSeq PCR-free library prep kit (Illumina) and 2 × 150 bp paired-end reads were sequenced on a NovaSeq 6000 (Illumina) with a median coverage of 81–99×. The RNA-seq libraries were prepared with the Novogene NGS RNA Library Prep Set (PT042), and bisulfite-treated DNA libraries of whole-genome bisulfite sequencing (WGBS) were sequenced on a NovaSeq X Plus. Sequencing data were processed and analyzed using standard pipelines. Detailed protocols, including library preparation and data analysis workflows, are provided in Additional file 2: Supplementary Methods.

### ABCB1 expression analysis

Total RNA was isolated from cell pellets using the AllPrep DNA/RNA kit (Qiagen GmbH, Hilden, Germany) following the manufacturer’s protocol. After quantification, 1 µg of RNA was reverse transcribed with SuperScript IV VILO Mastermix (Thermo Fisher Scientific GmbH, Dreieich, Germany) and quantitative PCR (qPCR) was performed on a StepOnePlus using TaqMan™ Gene Expression Master Mix, Hs00184500_m1 for *ABCB1* and Hs00939627_m1 for endogenous control *GUSB*. The 2^(-∆∆CT)^ approach was applied to compute relative quantification.

For protein quantification, frozen cell pellets were lysed in ice-cold RIPA buffer containing Halt protease inhibitor (Thermo Fisher Scientific GmbH, Dreieich, Germany). Lysates were denatured in Laemmli buffer, subjected to SDS-PAGE, and finally transferred to nitrocellulose membranes. Membranes were incubated overnight at 4 °C with primary antibodies against ABCB1 (Cell Signaling Technology #13987, 1:10,000) and GAPDH (Cell Signaling Technology #2118, 1:5,000), and subsequently incubated with HRP-conjugated secondary antibody (Cell Signaling Technology #7074, 1:10,000). Detection was performed using ECL substrate (Bio‑Rad Laboratories GmbH, Feldkirchen, Germany), and images were acquired with a ChemiDoc Imaging system (Bio‑Rad Laboratories GmbH, Feldkirchen, Germany).

### Targeted bisulfite sequencing

500 ng of genomic DNA was bisulfite-converted using the EpiTect^®^ Bisulfite Kit (Qiagen GmbH, Hilden, Germany). The *ABCB1* promoter region (GRCh38:7:87600603–87600971; strand: − 1) was PCR-amplified using the forward (5´-TGAGGTTGATTGGTTGGGTAGGAATA) and reverse (5´-TAACCCCACTTAATCCCCATAAACTTACC) primers. PCR products were gel-purified and subjected to Sanger sequencing on an ABI DNA Analyzer. CpG site methylation was analyzed using SnapGene software (v.5.0.8) [21].

### Dual-luciferase reporter assay

The *ABCB1* downstream promoter region containing the transcription start site (GRCh38:7:87600603–87600971:-1) was cloned into the CpG-free pCpGL vector [19], and transformed into PIR1 One-Shot Competent Cells (Thermo Fisher Scientific GmbH, Dreieich, Germany). Following colony PCR confirmation, positive clones were cultured overnight, and plasmid DNA was purified and *in vitro *methylated using M.SssI methyltransferase (New England Biolabs GmbH, Frankfurt am Main, Germany). Methylated or unmethylated pCpGL-*ABCB1* constructs were co-transfected with Renilla and CD4 vectors into AMO1 or SH-SY5Y cells. CD4-positive cells were selected via magnetic bead separation, and luciferase activity was measured using a Tecan Infinite 200 Pro plate reader (Tecan Deutschland GmbH, Crailsheim, Germany).

### Statistical analysis

Statistical comparisons between two groups were performed using either the unpaired t-test (for normally distributed data) or the Mann-Whitney U test (for non-parametric data). Associations between *ABCB1* promoter methylation and *ABCB1* expression were evaluated by Spearman correlation.* p* values < 0.05 were considered statistically significant. All statistical analyses were conducted using GraphPad Prism software (v.10.1.2).

## Results

### WGS identifies proteasome-related alterations but no genomic changes at the *ABCB1* locus in CFZ-resistant myeloma

**Fig. 1:**
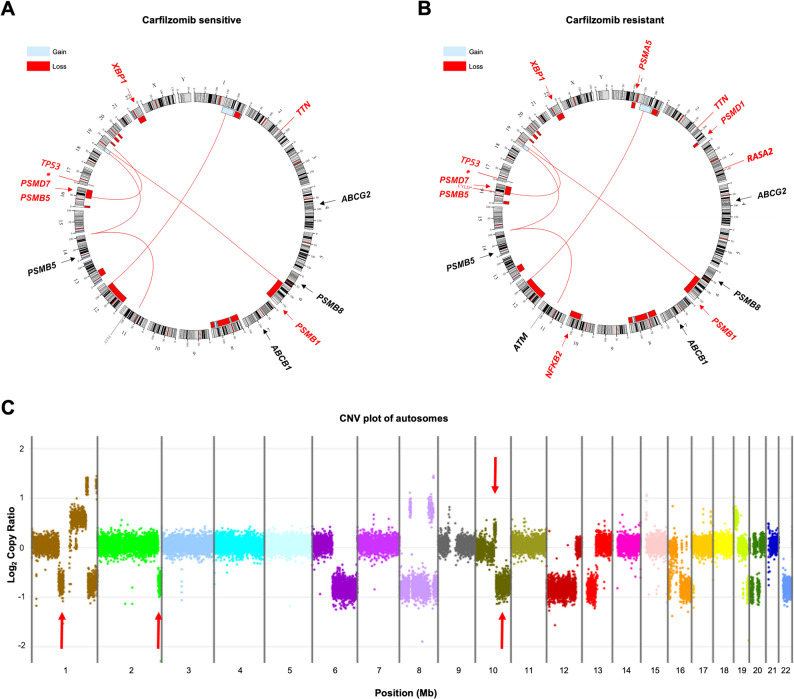
Whole-genome sequencing of paired CFZ-sensitive and CFZ-resistant patient samples. **A**, **B** Circos plots with copy number variations (CNVs), interchromosomal structural variations (SVs), and single-nucleotide variants (SNVs) based on WGS data before therapy with carfilzomib and at the time of resistance to carfilzomib. Outer track runs clockwise from chromosome 1 to Y. Inner track shows CNVs (gains in light blue and losses in red). Red lines inside the circle represent interchromosomal SVs. Outside of the circle, genes with an SNV (Tier 1 or Tier 2) are depicted in red and genes with a variant of unknown significance (VUS) are shown in gray. On the very outside, the chromosomal localization of specific genes that could be relevant for carfilzomib resistance is indicated by arrows (in red in the case of a deletion). **C** CNV plot of autosomes 1–22. Red arrows highlight gains and losses identified in the carfilzomib-resistant sample compared to WGS data of a sample prior to carfilzomib treatment

To investigate whether genomic alterations underlie *ABCB1* upregulation or reveal additional contributors to resistance, we performed whole-genome sequencing (WGS) on paired samples from a patient previously described by Besse et al. [14]. This patient exhibited a marked increase in *ABCB1* expression upon developing CFZ resistance. We compared genomic profiles obtained before treatment (CFZ sensitive) and after the development of resistance (CFZ resistant) using WGS data (Fig. 1A, B, circos plot). Two large deletions were acquired in the resistant sample: an ~ 18.8 Mb deletion in the 1p21.2–p12 region (chr1:101684001–120532000) affecting the *PSMA5* gene (mean log_2_ ratio: − 0.70), and a ~ 13.4 Mb deletion in the 2q36.3–q37.3 region (chr2:229817001–243185000) encompassing the *PSMD1* gene (mean log_2_ ratio: − 0.70). Of note, the identified 2q deletion also includes the genes *COPS7B* and *COPS8*, both belonging to the COP9 signalosome family for which an association with resistance to immunomodulatory drugs (IMiDs) has been described [22]. Beyond these deletions, additional copy number variations (CNVs) were observed on chromosome 10q comprising a ~ 7.6 Mb gain at 10q22.1–q22.3 (mean log_2_ ratio: + 0.35) adjacent to a large deletion of ~ 56.8 Mb at 10q22.3–q26.3 (mean log_2_ ratio: − 0.73) encompassing the *NFKB2* gene (Additional file 1: CNV). At the single nucleotide level, the resistant sample acquired two new mutations: a Tier 1 pathogenic mutation in the tumor suppressor gene *CYLD*, previously linked to unfavorable outcomes in MM, and a Tier 2 likely pathogenic mutation in *RASA2* (Additional file 1: SNV). Structural variation (SV) analysis showed consistent interchromosomal alterations in both samples including the translocation t(11;14)(q13;q32), but their variant allele frequencies were increased in the resistant sample (Additional file 1: SV), indicating clonal expansion. Importantly, despite genomic changes, our focused analysis of known proteasome inhibitor/CFZ resistance-associated genes (including *ABCB1*, *ABCG2*, *PSMB5*, *PSMB8*, *NFKB2*, and *XBP1* [23]) revealed no biallelic events at these loci. This suggests that while resistant cells undergo genomic rearrangements involving proteasome-related genes such as *PSMA5*, *PSMD1*,* NFKB2,* and *XBP1*, the substantial increase in *ABCB1* expression is likely regulated by non-genomic mechanisms.

### Transcriptomic profiling reveals *ABCB1* overexpression alongside epigenetic regulator upregulation in CFZ-resistant myeloma

**Fig. 2:**
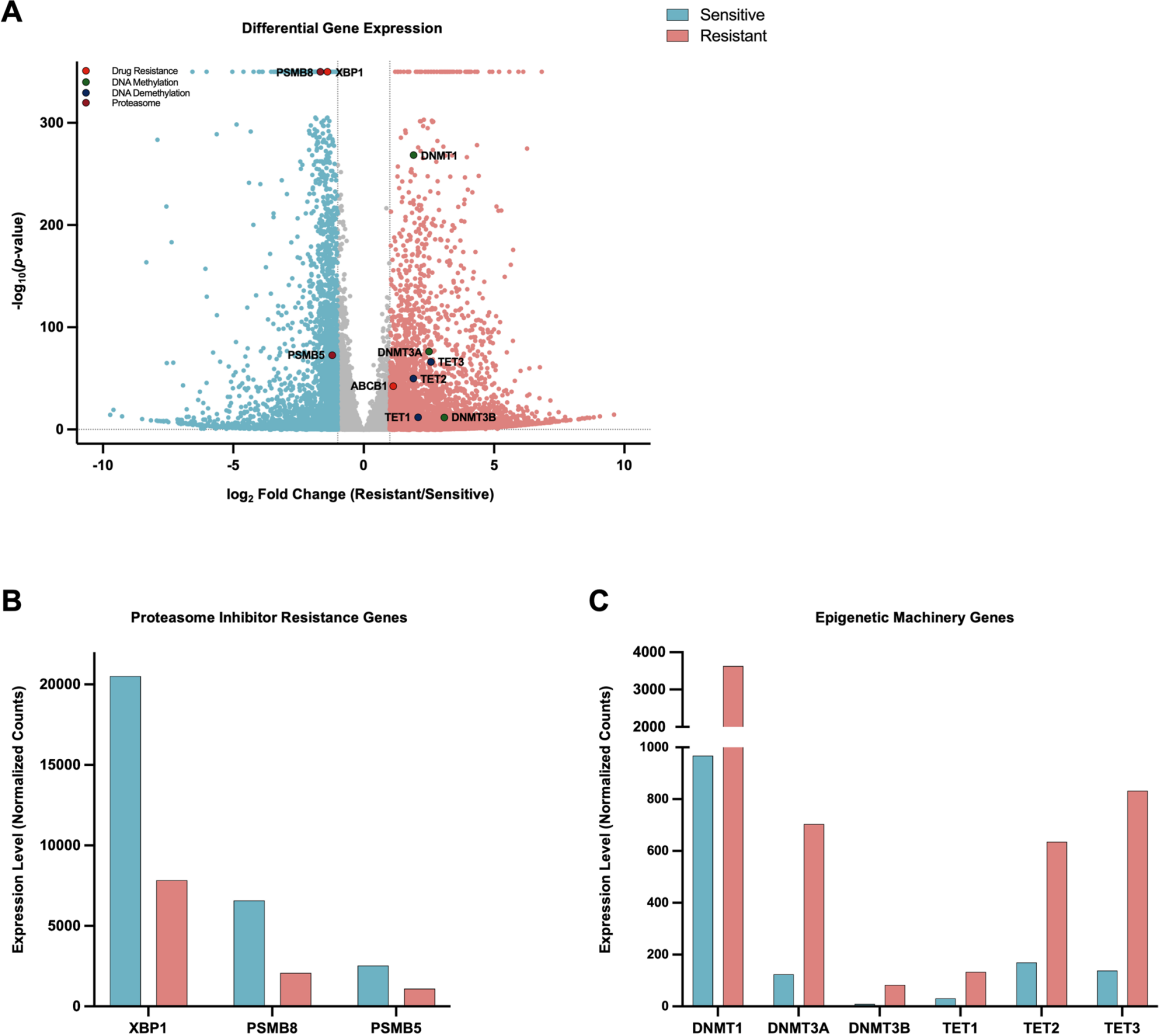
RNA-seq of paired CFZ-sensitive and CFZ-resistant patient samples. **A** Volcano plot displaying differential gene expression between carfilzomib-resistant and -sensitive patient samples. X-axis represents log2 fold change and Y-axis the -log10(adjusted p-value). Significantly differentially expressed genes are highlighted in colors based on functional categories: DNA methylation (green), drug resistance (orange), DNA demethylation (blue), and proteasome subunits (red). **B** Bar plot comparing expression levels of PI resistance–associated genes between carfilzomib-sensitive (blue) and -resistant (red) samples. **C** Bar plot showing expression of epigenetic regulator genes (DNA methyltransferases and DNA demethylating enzymes) in carfilzomib-sensitive (blue) and -resistant (red) samples.

To explore potential non-genomic mechanisms underlying *ABCB1* upregulation, we performed RNA-seq analysis on the paired CFZ-sensitive and CFZ-resistant patient samples. *ABCB1* expression was significantly higher in the CFZ-resistant (mean expression: 1142.4) compared to the CFZ-sensitive patient sample (mean expression: 519.9), representing a 2.2-fold increase (adjusted *p*-value = 4.67 × 10^-43^). Beyond *ABCB1*, the most upregulated genes included *FBXL7* and *INSRR* (log_2_FC ranging from 8.8 to 9.6), whereas the most downregulated included *IGLV3-1*, *CD24*, and *IGKV1-39* (log_2_FC ranging from − 9.7 to − 9.3) (Fig. 2A). Among proteasome inhibitor resistance–associated genes, we observed significant downregulation of *PSMB5*, *PSMB8*, and *XBP1* (log_2_FC between − 1.2 and − 1.7), suggesting a diminished reliance on proteasome function in the resistant sample (Fig. 2B). Notably, key modulators of the epigenetic machinery were robustly induced, with DNA methyltransferases (*DNMT1*, *DNMT3A*, *DNMT3B*) showing log_2_FC values between 1.9 and 3.1, and DNA demethylating enzymes (*TET1*, *TET2*, *TET3*) exhibiting log_2_FC values between 1.9 and 2.6 (Fig. 2C). Taken together, while *ABCB1* upregulation occurs in the absence of detectable genomic alterations, coordinated expression of epigenetic regulators suggests active remodeling of the epigenetic landscape, which likely contributes to the transcriptional upregulation of *ABCB1* and the overall resistant phenotype.

### Epigenetic profiling reveals reduced *ABCB1* promoter methylation after developing CFZ resistance

**Fig. 3:**
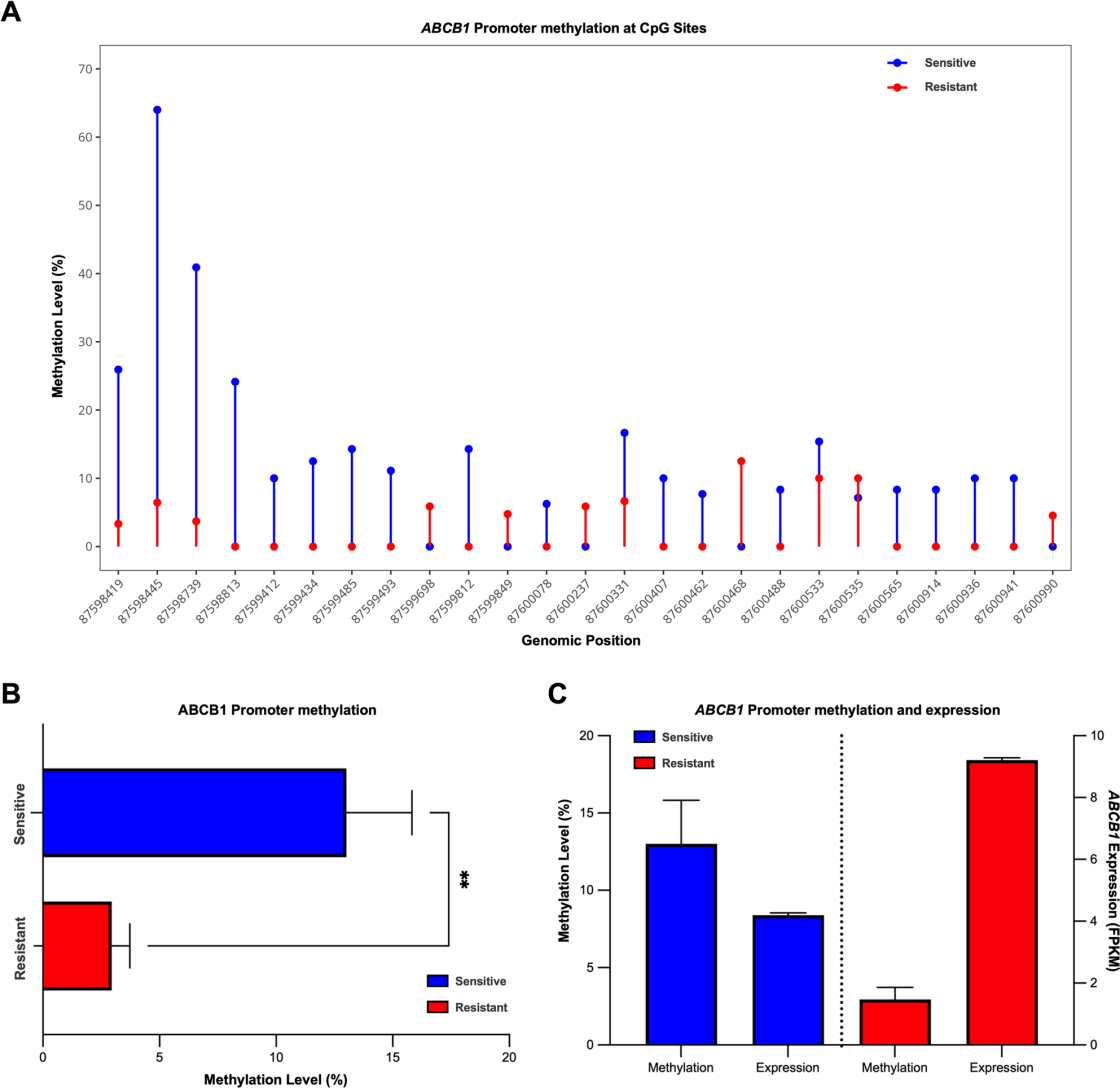
*ABCB1* promoter methylation and gene expression in paired CFZ-sensitive and CFZ-resistant samples.** A** Methylation profile of the canonical *ABCB1* promoter region (GRCh38:7:87598323–87601474) comparing carfilzomib-sensitive (blue) and -resistant (red) samples from the index patient. The plot displays site-specific CpG methylation levels across the promoter region.** B** Bar plot showing mean methylation levels across the entire *ABCB1* promoter region in carfilzomib-sensitive (blue) and -resistant (red) samples.** C** Comparison of *ABCB1* expression levels (FPKM) between carfilzomib-sensitive (blue) and -resistant (red) samples, demonstrating the negative association between promoter methylation and gene expression

To elucidate the potential epigenetic mechanisms underlying this upregulation, WGBS was conducted, and subsequent analyses focused on the canonical *ABCB1* promoter region (GRCh38:7:87598323–87601474). This focused evaluation demonstrated pronounced CpG hypomethylation in the CFZ-resistant sample relative to its sensitive counterpart. Site-specific methylation analysis revealed substantial decreases at the majority of the CpG sites, with methylation levels decreasing from 25.9 to 3.3% at position 87598419 and from 64 to 6.5% at position 87598445, representing absolute differences of 22.6 and 57.6%, respectively (Fig. 3A). Notably, across the promoter region, the sensitive sample exhibited a mean methylation level of 11.9% (± 2.4%; range: 0–64.0%), whereas the resistant sample displayed only 4.0% (± 1.3%; range: 0–34.6%) (Fig. 3B). An inverse relationship was observed between ABCB1 promoter methylation (11.9% in sensitive vs. 4.0% in resistant) and its gene expression (4.2 FPKM in sensitive vs. 9.2 FPKM in resistant) (Fig. 3C). To extend clinical relevance, an independent MM subcohort (*n* = 13) was analyzed for *ABCB1* promoter methylation and *ABCB1* expression. An inverse association was observed (Spearman ρ = − 0.59, *p* = 0.036), with promoter methylation ranging from 0.3 to 34.8% and *ABCB1* expression of 0.06–7.49 (log_2_[TPM + 1]). Notably, the highest expression was only observed in samples with < 1% promoter methylation (Additional file 2: Table S1 and Fig. S1). Together, these data indicate that the methylation of the *ABCB1* promoter regulates the transcriptional activity of *ABCB1*, enabling enhanced CFZ efflux and subsequent resistance in MM.

### Validation of epigenetic reprogramming-driven ABCB1-mediated carfilzomib resistance in MM cell line models

**Fig. 4:**
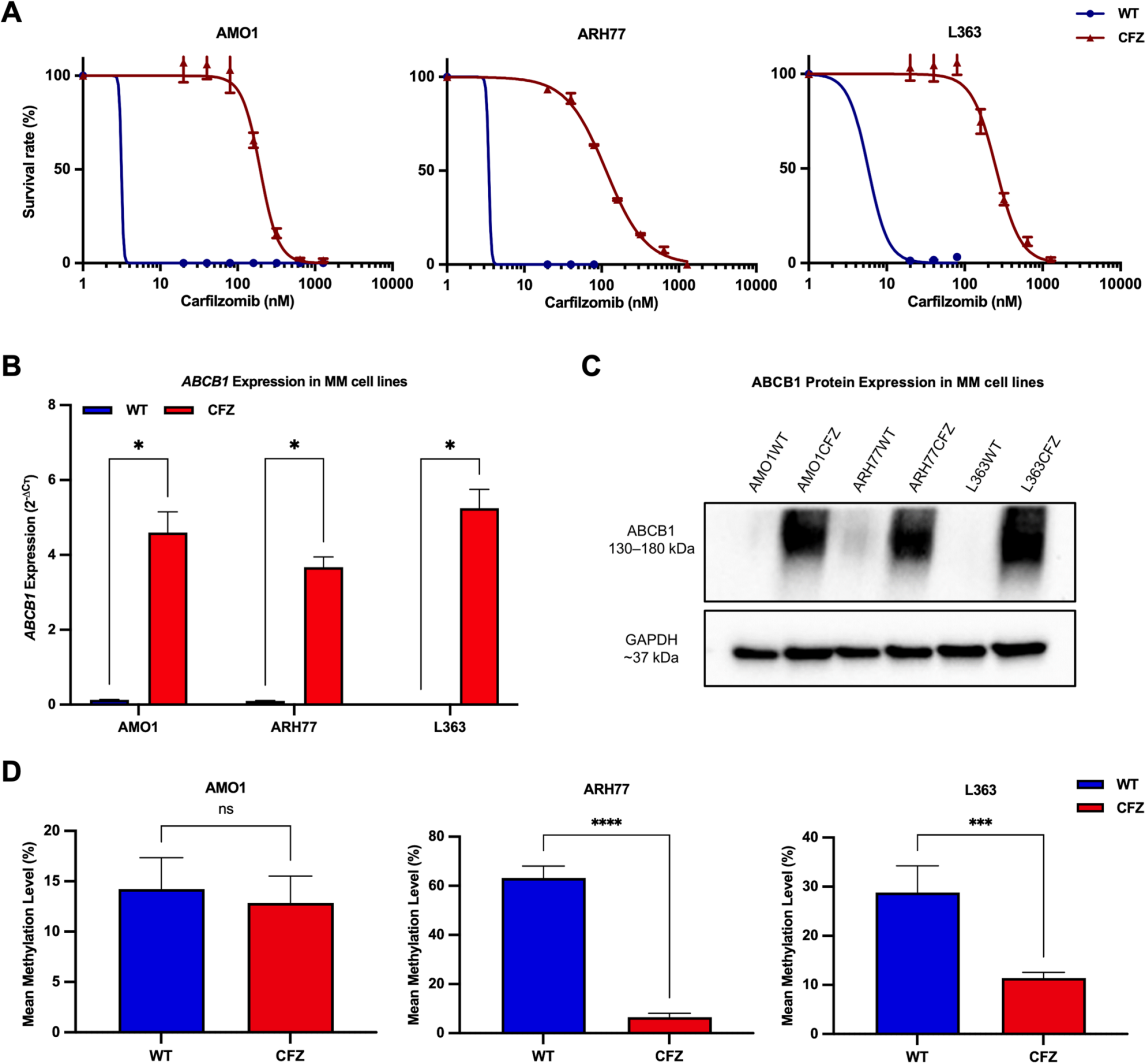
Functional validation of ABCB1 upregulation and promoter hypomethylation in CFZ-resistant MM cell lines.** A** Dose-response curves showing carfilzomib cytotoxicity in WT and resistant MM cell lines (AMO1, L363, and ARH77), with corresponding IC₅₀ values demonstrating significant resistance in CFZ-resistant derivatives.** B** Bar plot comparing *ABCB1* mRNA expression levels (2^(−ΔCT)^) between WT and CFZ-resistant cell lines, showing consistent upregulation across all three models.** C** Western blot analysis of ABCB1 protein expression in WT and CFZ-resistant cell lines, confirming transcriptional findings at the protein level.** D** Bar plot displaying *ABCB1* promoter methylation levels (GRCh38:7:87598323–87601474) in WT and CFZ-resistant cell lines, demonstrating significant hypomethylation in L363CFZ and ARH77CFZ models

To extend our patient-derived findings and further dissect the mechanisms of carfilzomib resistance, three independent CFZ-resistant MM cell line models (AMO1CFZ, L363CFZ, and ARH77CFZ) were generated through long-term exposure to escalating drug concentrations. Characterization via alamarBlue assays revealed that all CFZ-resistant cell lines exhibited significantly elevated IC_50_ values relative to their parental counterparts. AMO1CFZ cells displayed a 62.1-fold increase in IC_50_ (from 3.2 nM in WT to 195.7 nM in the resistant derivative). Similarly, L363CFZ cells showed a 43.8-fold increase (from 5.8 to 253.6 nM), while ARH77CFZ cells demonstrated a 41.5-fold increase (from 3.5 to 114.4 nM) (Fig. 4A).

Consistent with the patient data, expression analysis using qPCR demonstrated significant upregulation of *ABCB1* mRNA across all three CFZ-resistant cell lines. Quantitative analysis by the 2^(−ΔCT)^ method revealed that all WT cell lines exhibited low baseline *ABCB1* expression, with AMO1 and ARH77 showing modest levels (2^(−ΔCT)^ values of 0.13 and 0.1, respectively) and L363 displaying near-undetectable transcript levels (2^(−ΔCT)^ = 6.3 × 10^-6^). In contrast, all three CFZ-resistant derivatives demonstrated substantially elevated absolute expression levels (AMO1CFZ: 4.6; ARH77CFZ: 3.7; L363CFZ: 5.3). The comparison of these absolute 2^(−ΔCT)^ values clearly demonstrated a significant and consistent upregulation of *ABCB1* expression upon the development of carfilzomib resistance, with the L363 model highlighting the remarkable dynamic range of this regulatory mechanism (Fig. 4B). In parallel, Western blot analysis confirmed the transcriptomic findings at the protein level, demonstrating corresponding increases in ABCB1 protein expression compared to their WT counterparts (Fig. 4C), with strong concordance between mRNA and protein levels, indicating that the observed upregulation is primarily driven by transcriptional activation rather than post-translational mechanisms.

To further explore the regulatory underpinnings of *ABCB1* overexpression, WGBS with 30× coverage was performed on paired parental and CFZ-resistant cell lines, focusing specifically on the downstream promoter GH07J087598 (GRCh38:7:87598323–87601474). While the AMO1 models showed only marginal changes (14.2% vs. 12.9%), in the L363 and ARH77 models, the *ABCB1* downstream promoter exhibited methylation levels of 28.8 and 63.2% in WT cells, which decreased significantly to 11.4% (*p* = 0.0007) and 6.5% (*p* < 0.0001) in their resistant derivatives, respectively (Fig. 4D). Notably, a negative correlation was observed between promoter methylation and *ABCB1* expression in the L363 and ARH77 models, wherein substantial decreases in methylation corresponded with pronounced increases in *ABCB1* expression. This pattern, which closely mirrors the epigenetic alterations observed in patient specimens, supports a conserved mechanism driving *ABCB1* overexpression under CFZ-induced selective pressure.

### Functional validation confirms methylation-dependent regulation of *ABCB1* expression

**Fig. 5:**
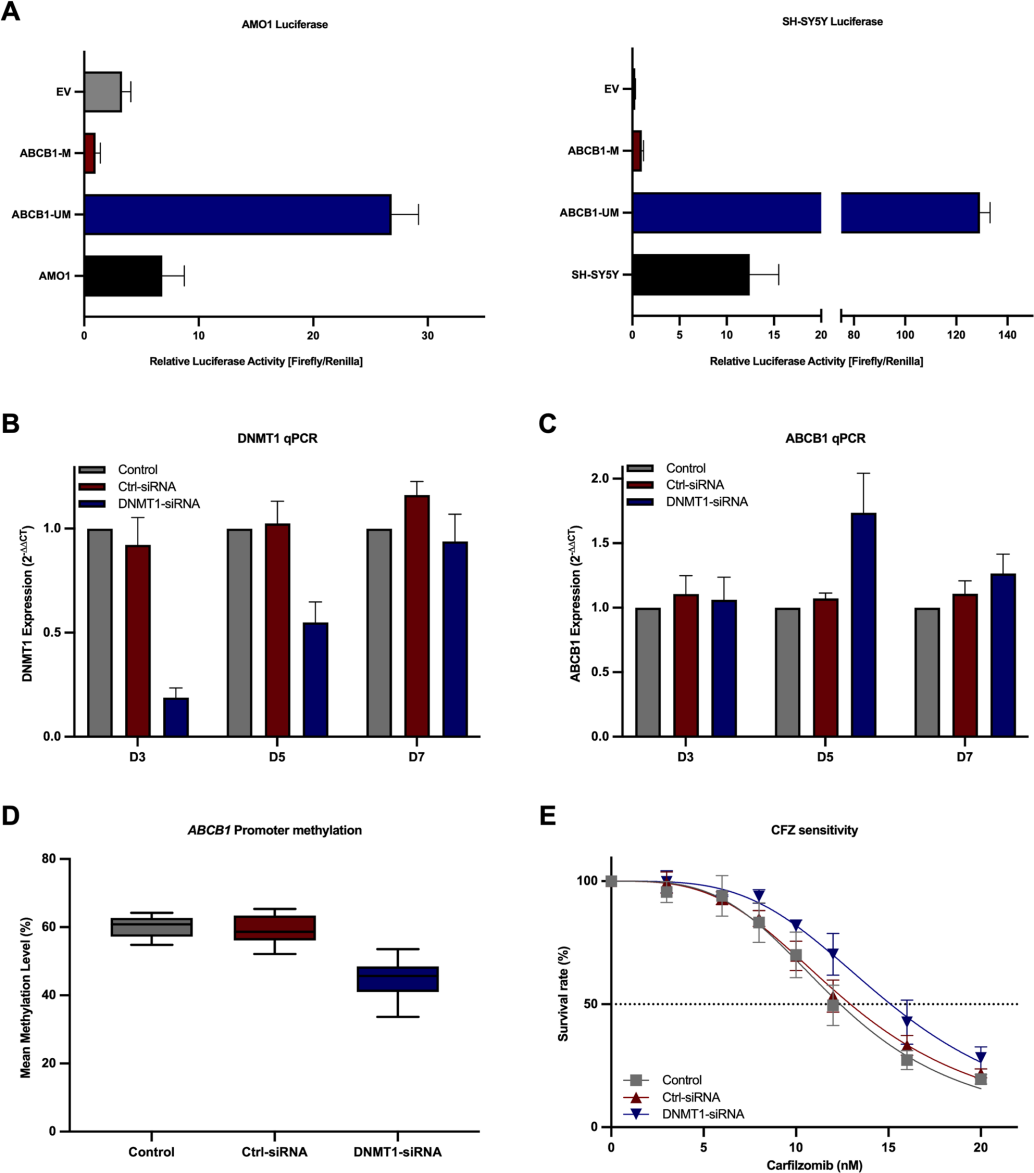
Methylation-dependent regulation of *ABCB1* promoter activity and expression in *in vitro* models. **A** Dual-luciferase reporter assay showing *ABCB1* promoter activity in AMO1 and SH-SY5Y cells with unmethylated (ABCB1-UM) or methylated (ABCB1-M) pCpGL-*ABCB1* constructs, or the empty vector control (EV), demonstrating methylation-dependent transcriptional repression. **B** *DNMT1* expression (qPCR) of DNMT1 siRNA (blue), non-targeting siRNA (Ctrl-siRNA, red), and electroporation control (gray). **C** *ABCB1* expression (qPCR) of DNMT1 siRNA (blue), non-targeting siRNA (Ctrl-siRNA, red), and electroporation control (gray). **D** *ABCB1* promoter methylation level (%) of electroporation control (gray), non-targeting siRNA (Ctrl-siRNA, red), and DNMT1 siRNA (blue) at day 5. **E** CFZ sensitivity of DNMT1 siRNA (blue), non-targeting siRNA (Ctrl-siRNA, red), and electroporation control (gray)

To investigate whether promoter methylation regulates *ABCB1* expression, we conducted a dual-luciferase reporter assay to assess promoter activity, and subsequently treated cells with DNA methyltransferase inhibitors (DNMTis) to evaluate effects on endogenous gene expression and methylation. First, the impact of methylation on the *ABCB1* downstream promoter (GH07J087598) was evaluated using a CpG-free vector system (pCpGL). In AMO1 cells, the normalized luciferase activity (Firefly/Renilla) of the unmethylated pCpGL-*ABCB1* construct was 26.8-fold higher than that of the empty vector, confirming the promoter functionality of the cloned *ABCB1* core region. Methylation of the construct reduced luciferase activity by 8.1-fold, clearly demonstrating methylation-dependent transcriptional repression. Similarly, in SH-SY5Y cells, methylation of the *ABCB1* promoter construct led to a 129.3-fold decrease in activity relative to the unmethylated version, further supporting that hypermethylation significantly impairs the transcriptional capacity of the *ABCB1* downstream promoter (Fig. 5A). These results confirm that hypermethylation of the promoter directly impairs its activity.

To further examine this relationship in a cellular context, ARH77WT cells, characterized by high baseline *ABCB1* promoter methylation (63.2%), were treated with two DNMTis (25 nM decitabine and 1 µM GSK-3484862). After seven days of treatment, site-specific analysis revealed that both DNMTi treatments consistently reduced methylation across all individual CpG sites within the *ABCB1* core promoter region compared to the control, demonstrating a comprehensive demethylation effect (Additional file 2: Fig. S2A). This pattern was reflected in overall promoter methylation levels: DMSO-treated control cells maintained high methylation (69.9%), while decitabine reduced methylation to 38%, and GSK-3484862 further decreased it to 19.7% (Additional file 2: Fig. S2B). Notably, *ABCB1* expression (measured by 2^(−ΔΔCT)^) increased significantly with a 21.7-fold upregulation following decitabine treatment and a 35.7-fold increase with GSK-3484862 (Additional file 2: Fig. S2C), which inversely correlated with the observed promoter hypomethylation. However, DNMTi exposure decreased cell viability over time (Additional file 2: Fig. S2D) and co-treatment with sub-cytotoxic GSK concentrations (500 nM, 1 µM) and CFZ (72 h) showed no detectable shift in the CFZ IC_50_ relative to DMSO (Additional file 2: Fig. S2E).

To avoid DNMTi-related cytotoxicity, we next performed DNMT1 KD using siRNAs. ARH77WT cells were electroporated with a DNMT1 siRNA or a non-targeting control siRNA (Ctrl-siRNA). The mRNA level of *DNMT1* was lowest at day 3 and partially recovered over time (Fig. 5B), whereas *ABCB1* expression level peaked at day 5 and declined by day 7 (Fig. 5C). In parallel, *ABCB1* promoter methylation was significantly reduced under DNMT1 KD at day 5 (44.8%) compared with both controls (59.3–60.1%) (Fig. 5D). In the following CFZ sensitivity test at day 5, DNMT1 KD cells revealed a right-shift in the CFZ IC_50_ (15.12 nM) relative to the CFZ IC_50_ from electroporation control (12.39 nM) and Ctrl-siRNA (12.94 nM) (Fig. 5E), indicating reduced CFZ sensitivity.

Together, these data demonstrate that reducing or inhibiting DNMT1 lowers *ABCB1* promoter methylation levels and restores *ABCB1* expression, supporting a methylation-dependent regulatory mechanism that contributes to CFZ resistance in MM cells.

## Discussion

Genetic and epigenetic alterations in key therapeutic targets contribute significantly to drug resistance in MM. For instance, *CRBN*-coding mutations impair the binding of IMiDs [24], while hypermethylation of an intronic *CRBN* enhancer can silence its expression [25]. In the context of GPRC5D-directed immunotherapy, resistance has been linked to inactivating mutations/deletions [26, 27] and/or hypermethylation of the *GPRC5D* promoter [28]. Comparable epigenetic and genetic mechanisms have been implicated in resistance to proteasome inhibitors. Mutations in the proteasome subunit beta type-5 (*PSMB5*) alter proteasome conformation, reducing chymotrypsin-like activity and impairing PI binding. However, such mutations are rarely detected in patients and are mostly confined to cell line models, limiting their clinical relevance [29]. More compelling is the observation that altered promoter methylation of *PSMD5* is present in approximately 25% of PI-refractory patients [13].

Beyond proteasome subunit alterations, increased expression of drug efflux pumps also plays a critical role. The multidrug resistance transporter ABCB1, in particular, is upregulated in both PI-resistant patient samples and CFZ-resistant cell line models. Importantly, HIV protease inhibitors such as nelfinavir and lopinavir have been shown to inhibit ABCB1 activity, thereby restoring carfilzomib’s proteasome-inhibitory function and overcoming drug resistance at clinically achievable concentrations [14]. However, the molecular mechanisms driving *ABCB1* upregulation remain largely unclear. While genetic alterations have been proposed to influence *ABCB1* expression [30], the accumulating evidence points to a significant role for epigenetic regulation in modulating *ABCB1* levels and contributing to treatment resistance across various cancer types [31–33]. This is further supported by the presence of a distinct downstream *ABCB1* promoter (GH07J087598; GRCh38:7:87598323–87601474), in which differential methylation can modulate its transcriptional activity directly and may also facilitate transcription factor (TF) binding [34, 35]. In this context, NF-κB, AP-1, and FOXP1 have been reported to regulate *ABCB1* expression in other malignancies [36–38].

Building on this knowledge, we analyzed the methylation status of this downstream *ABCB1* promoter and identified consistent hypomethylation in a primary CFZ-resistant MM patient sample. This was associated with pronounced *ABCB1* upregulation in the absence of genetic mutations or copy number alterations. In addition, transcriptomic profiling revealed elevated expression of several key epigenetic regulators, including DNMTs and TET family enzymes, suggesting broad epigenetic reprogramming during the development of resistance. Consistent with the paired CFZ-resistant patient case, an independent MM subcohort showed an inverse association between *ABCB1* promoter methylation and *ABCB1* expression. Although this cohort was not enriched for CFZ-resistant cases, the observed pattern supports the clinical relevance of *ABCB1* promoter regulation in MM.

This methylation pattern was recapitulated in our CFZ-resistant cell line models, which showed pronounced promoter hypomethylation together with *ABCB1* upregulation compared to their parental CFZ-sensitive WTs, mirroring the paired patient case. Functional validation with dual-luciferase assays confirmed that methylation of GH07J087598 directly suppresses promoter activity. Furthermore, pharmacologic inhibition of DNMT1 hypomethylated the *ABCB1* promoter, and DNMT1 KD reduced promoter methylation while inducing *ABCB1* expression. KD of DNMT1 also shifted the CFZ IC_50_ to higher concentrations, supporting a model in which promoter hypomethylation induces *ABCB1* expression, thereby increasing efflux of CFZ and reducing CFZ sensitivity.

Given that CFZ is an ABCB1 substrate, DNMTi-mediated upregulation of ABCB1 would be expected to antagonize CFZ. By contrast, decitabine has been reported to restore BTZ sensitivity [39], for which other resistance mechanisms have been described (e.g., *PSMB5* mutations, altered proteasome assembly, or *PSMD5* hypermethylation) [13, 29]. In our DNMTi experiments, concentrations required to demethylate the *ABCB1* promoter were cytotoxic. However, the CFZ IC_50_ did not shift, likely reflecting a balance between modest *ABCB1* upregulation and DNMTi cytotoxicity. This was supported by the DNMT1 KD experiment which confirmed ABCB1-mediated antagonism to CFZ.

Another layer of transcriptional regulation is provided by histone modifications. Prior studies indicate that histone acetylation at the *ABCB1* promoter can enhance transcription and promote drug resistance in non-MM cancer models [40, 41]. While our data establish promoter hypomethylation as a putative driver of ABCB1 activation in CFZ-resistant MM, the specific histone marks and transcription factors that may cooperate with DNA hypomethylation at this locus remain to be defined to fully understand the epigenetic regulation of *ABCB1*. Methodologically, our study comprises a paired CFZ-resistant case, CFZ-resistant cell-line models, and a small independent MM subcohort. Thus, larger, longitudinal CFZ-treated cohorts are required to determine the incidence and clinical outcome of *ABCB1* promoter hypomethylation in MM. Finally, while *ABCB1* overexpression is clearly an important factor, it is likely that other resistance mechanisms also contribute to the complex phenotype observed in CFZ-resistant MM. Expanding the understanding of these additional mechanisms will be crucial for developing comprehensive strategies to overcome resistance and improve treatment outcomes.

In summary, our findings identify hypomethylation of a downstream *ABCB1* promoter as a previously unrecognized but functionally important mechanism of carfilzomib resistance in MM. These results highlight DNA methylation as a potentially actionable target and support further exploration of epigenetic therapies to overcome PI resistance in MM.

## Supplementary Information

Below is the link to the electronic supplementary material.

Additional file 1 (.xlsx). Whole-genome sequencing results of paired CFZ-sensitive and CFZ-resistant patient samples. CNVs (copy number variations called using GATK); SNVs (single nucleotide variants and small indels called with Strelka); SVs (structural variants called with Manta). Variants are listed after filtering against gnomAD (v.2.1.1) at >0.05% global population frequency.

Additional file 2 (.docx). Supplementary Information. Table S1 (clinical annotation for the independent MM subcohort, n = 13); Figure S1 (scatter plot of ABCB1 promoter methylation vs. expression in the independent MM subcohort); Figure S2 (DNMTi treatment results in ARH77WT cells); and Supplementary Methods.

Additional file 3 (.pdf). Uncropped Western blot images for Figure 4C showing ABCB1 and GAPDH protein expression in WT and CFZ-resistant MM cell lines.

## Data Availability

The anonymized methylation data from WGBS are available in the Zenodo repository with the dataset identifier (DOI: 10.5281/zenodo.18547283). The raw whole-genome sequencing (WGS), whole-genome bisulfite sequencing (WGBS), and RNA sequencing data generated from the patient samples are not publicly available in accordance with European General Data Protection Regulation (GDPR). Access to the raw genome-wide sequencing data can be provided upon reasonable request to the corresponding author, subject to ethical approval and a Data Transfer Agreement.

## Abbreviations

BTZ: Bortezomib
CFZ: Carfilzomib
CNV: Copy number variation
Dec: Decitabine
DMSO: Dimethyl sulfoxide
DNMTi: DNA methyltransferase inhibitor
FC: Fold change
FPKM: Fragments per kilobase of transcript per million mapped reads
GSK: GSK-3484862
IMiDs: Immunomodulatory drugs
IC_50_: Half-maximal inhibitory concentration
KD: Knockdown
MM: Multiple myeloma
PI: Proteasome inhibitor
qPCR: Quantitative polymerase chain reaction
RNA-seq: RNA sequencing
SNV: Single nucleotide variant
STR: Short tandem repeat
SV: Structural variant
TPM: Transcript per million
WGBS: Whole-genome bisulfite sequencing
WGS: Whole-genome sequencing
WT: Wild-type

## References

[1] Rajkumar SV. Multiple myeloma: 2024 update on diagnosis, risk-stratification, and management. Am J Hematol. 2024;99(9):1802–24. 38943315 10.1002/ajh.27422 PMC11404783

[2] Kumar SK, Rajkumar V, Kyle RA, van Duin M, Sonneveld P, Mateos MV, et al. Multiple myeloma. Nat Rev Dis Primers. 2017;3:17046. 28726797 10.1038/nrdp.2017.46

[3] McConkey DJ, Zhu K. Mechanisms of proteasome inhibitor action and resistance in cancer. Drug Resist Updat. 2008;11(4–5):164–79. 18818117 10.1016/j.drup.2008.08.002

[4] Wang J, Fang Y, Fan RA, Kirk CJ. Proteasome inhibitors and their pharmacokinetics, pharmacodynamics, and metabolism. Int J Mol Sci. 2021;22(21):11595. 34769030 10.3390/ijms222111595 PMC8583966

[5] Gandolfi S, Laubach JP, Hideshima T, Chauhan D, Anderson KC, Richardson PG. The proteasome and proteasome inhibitors in multiple myeloma. Cancer Metastasis Rev. 2017;36(4):561–84. 29196868 10.1007/s10555-017-9707-8

[6] Dima D, Jiang D, Singh DJ, Hasipek M, Shah HS, Ullah F, et al. Multiple myeloma therapy: emerg trends challenges cancers. 2022;14(17):4082. 10.3390/cancers14174082 PMC9454959 36077618

[7] Anderson KC. Progress and paradigms in multiple myeloma. Clin Cancer Res. 2016;22(22):5419–27. 28151709 10.1158/1078-0432.CCR-16-0625 PMC5300651

[8] Weir P, Donaldson D, McMullin MF, Crawford L. Metabolic alterations in multiple myeloma: from oncogenesis to proteasome inhibitor resistance. Cancers. 2023;15(6):1682. 36980568 10.3390/cancers15061682 PMC10046772

[9] Ge M, Qiao Z, Kong Y, Liang H, Sun Y, Lu H, et al. Modulating proteasome inhibitor tolerance in multiple myeloma: an alternative strategy to reverse inevitable resistance. Br J Cancer. 2021;124(4):770–6. 33250513 10.1038/s41416-020-01191-y PMC7884794

[10] Niewerth D, Jansen G, Assaraf YG, Zweegman S, Kaspers GJ, Cloos J. Molecular basis of resistance to proteasome inhibitors in hematological malignancies. Drug Resist Updat. 2015;18:18–35. 25670156 10.1016/j.drup.2014.12.001

[11] Allmeroth K, Horn M, Kroef V, Miethe S, Muller RU, Denzel MS. Bortezomib resistance mutations in PSMB5 determine response to second-generation proteasome inhibitors in multiple myeloma. Leukemia. 2021;35(3):887–92. 32690882 10.1038/s41375-020-0989-4 PMC7932915

[12] Unno M, Mizushima T, Morimoto Y, Tomisugi Y, Tanaka K, Yasuoka N, et al. The structure of the mammalian 20S proteasome at 2.75 A resolution. Structure. 2002;10(5):609–18. 12015144 10.1016/s0969-2126(02)00748-7

[13] Haertle L, Barrio S, Munawar U, Han S, Zhou X, Simicek M, et al. Single-nucleotide variants and epimutations induce proteasome inhibitor resistance in multiple myeloma. Clin Cancer Res. 2023;29(1):279–88. 36282272 10.1158/1078-0432.CCR-22-1161

[14] Besse A, Stolze SC, Rasche L, Weinhold N, Morgan GJ, Kraus M, et al. Carfilzomib resistance due to ABCB1/MDR1 overexpression is overcome by nelfinavir and lopinavir in multiple myeloma. Leukemia. 2018;32(2):391–401. 28676669 10.1038/leu.2017.212 PMC5808083

[15] Sharom FJ. ABC multidrug transporters: structure, function and role in chemoresistance. Pharmacogenomics. 2008;9(1):105–27. 18154452 10.2217/14622416.9.1.105

[16] Byrgazov K, Kraus M, Besse A, Slipicevic A, Lehmann F, Driessen C, et al. Up-regulation of multidrug resistance protein MDR1/ABCB1 in carfilzomib-resistant multiple myeloma differentially affects efficacy of anti-myeloma drugs. Leuk Res. 2021;101:106499. 33422770 10.1016/j.leukres.2020.106499

[17] Spitzwieser M, Pirker C, Koblmuller B, Pfeiler G, Hacker S, Berger W, et al. Promoter methylation patterns of ABCB1, ABCC1 and ABCG2 in human cancer cell lines, multidrug-resistant cell models and tumor, tumor-adjacent and tumor-distant tissues from breast cancer patients. Oncotarget. 2016;7(45):73347–69. 27689338 10.18632/oncotarget.12332 PMC5341984

[18] Demidenko R, Razanauskas D, Daniunaite K, Lazutka JR, Jankevicius F, Jarmalaite S. Frequent down-regulation of ABC transporter genes in prostate cancer. BMC Cancer. 2015;15:683. 26459268 10.1186/s12885-015-1689-8 PMC4603841

[19] Klug M, Rehli M. Functional analysis of promoter CPG-methylation using a CpG-free luciferase reporter vector. Epigenetics. 2006;1(3):127–30. 17965610 10.4161/epi.1.3.3327

[20] Soriano GP, Besse L, Li N, Kraus M, Besse A, Meeuwenoord N, et al. Proteasome inhibitor-adapted myeloma cells are largely independent from proteasome activity and show complex proteomic changes, in particular in redox and energy metabolism. Leukemia. 2016;30(11):2198–207. 27118406 10.1038/leu.2016.102 PMC5097071

[21] Parrish RR, Day JJ, Lubin FD. Direct bisulfite sequencing for examination of DNA methylation with gene and nucleotide resolution from brain tissues. Curr Protoc Neurosci. 2012;60(1):7–24. 10.1002/0471142301.ns0724s60 PMC3395468 22752896

[22] Gooding S, Ansari-Pour N, Kazeroun M, Karagoz K, Polonskaia A, Salazar M, et al. Loss of COP9 signalosome genes at 2q37 is associated with IMiD resistance in multiple myeloma. Blood. 2022;140(16):1816–21. 35853156 10.1182/blood.2022015909 PMC10653034

[23] Leung-Hagesteijn C, Erdmann N, Cheung G, Keats JJ, Stewart AK, Reece DE, et al. Xbp1s-negative tumor B cells and pre-plasmablasts mediate therapeutic proteasome inhibitor resistance in multiple myeloma. Cancer Cell. 2013;24(3):289–304. 24029229 10.1016/j.ccr.2013.08.009 PMC4118579

[24] Kortum KM, Mai EK, Hanafiah NH, Shi CX, Zhu YX, Bruins L, et al. Targeted sequencing of refractory myeloma reveals a high incidence of mutations in CRBN and Ras pathway genes. Blood. 2016;128(9):1226–33. 27458004 10.1182/blood-2016-02-698092 PMC5524534

[25] Haertle L, Barrio S, Munawar U, Han S, Zhou X, Vogt C, et al. Cereblon enhancer methylation and IMiD resistance in multiple myeloma. Blood. 2021;138(18):1721–6. 34115836 10.1182/blood.2020010452 PMC8569411

[26] Ma S, Xia J, Zhang M, Li W, Xiao M, Sha Y, et al. Genetic and epigenetic mechanisms of GPRC5D loss after anti-GPRC5D CAR T-cell therapy in multiple myeloma. Blood. 2025;146(2):178–90. 40090012 10.1182/blood.2024026622 PMC12782979

[27] Derrien J, Gastineau S, Frigout A, Giordano N, Cherkaoui M, Gaborit V, et al. Acquired resistance to a GPRC5D-directed T-cell engager in multiple myeloma is mediated by genetic or epigenetic target inactivation. Nat Cancer. 2023;4(11):1536–43. 37653140 10.1038/s43018-023-00625-9

[28] Han S, Munawar U, Truger M, Gerhard-Hartmann E, Verbruggen C, Pfeiffer L et al. Promoter hypermethylation as a reversible mechanism of resistance to GPRC5D-directed therapy in multiple myeloma. Haematologica. 2026.10.3324/haematol.2025.28916241504227

[29] Barrio S, Stühmer T, Da-Viá M, Barrio-Garcia C, Lehners N, Besse A, et al. Spectrum and functional validation of PSMB5 mutations in multiple myeloma. Leukemia. 2019;33(2):447–56. 30026573 10.1038/s41375-018-0216-8

[30] Zawadzka I, Jelen A, Pietrzak J, Zebrowska-Nawrocka M, Michalska K, Szmajda-Krygier D, et al. The impact of ABCB1 gene polymorphism and its expression on non-small-cell lung cancer development, progression and therapy - preliminary report. Sci Rep. 2020;10(1):6188. 32277145 10.1038/s41598-020-63265-4 PMC7148348

[31] Reed K, Hembruff SL, Laberge ML, Villeneuve DJ, Côté GB, Parissenti AM. Hypermethylation of the ABCB1 downstream gene promoter accompanies ABCB1 gene amplification and increased expression in docetaxel-resistant MCF-7 breast tumor cells. Epigenetics. 2008;3(5):270–80. 19001875 10.4161/epi.3.5.6868

[32] Li A, Song J, Lai Q, Liu B, Wang H, Xu Y, et al. Hypermethylation of ATP-binding cassette B1 (ABCB1) multidrug resistance 1 (MDR1) is associated with cisplatin resistance in the A549 lung adenocarcinoma cell line. Int J Exp Pathol. 2016;97(6):412–21. 27995666 10.1111/iep.12212 PMC5370221

[33] Tomiyasu H, Goto-Koshino Y, Fujino Y, Ohno K, Tsujimoto H. Epigenetic regulation of the ABCB1 gene in drug-sensitive and drug-resistant lymphoid tumour cell lines obtained from canine patients. Vet J. 2014;199(1):103–9. 24332606 10.1016/j.tvjl.2013.10.022

[34] Stadler MB, Murr R, Burger L, Ivanek R, Lienert F, Scholer A, et al. DNA-binding factors shape the mouse methylome at distal regulatory regions. Nature. 2011;480(7378):490–5. 22170606 10.1038/nature10716

[35] Domcke S, Bardet AF, Adrian Ginno P, Hartl D, Burger L, Schubeler D. Competition between DNA methylation and transcription factors determines binding of NRF1. Nature. 2015;528(7583):575–9. 26675734 10.1038/nature16462

[36] Bentires-Alj M, Barbu V, Fillet M, Chariot A, Relic B, Jacobs N, et al. NF-kappaB transcription factor induces drug resistance through MDR1 expression in cancer cells. Oncogene. 2003;22(1):90–7. 12527911 10.1038/sj.onc.1206056

[37] Tsai YT, Lozanski G, Lehman A, Sass EJ, Hertlein E, Salunke SB, et al. BRAF(V600E) induces ABCB1/P-glycoprotein expression and drug resistance in B-cells via AP-1 activation. Leuk Res. 2015;39(11):1270–7. 10.1016/j.leukres.2015.08.017 PMC4779435 26350141

[38] Choi EJ, Seo EJ, Kim DK, Lee SI, Kwon YW, Jang IH, et al. FOXP1 functions as an oncogene in promoting cancer stem cell-like characteristics in ovarian cancer cells. Oncotarget. 2016;7(3):3506–19. 26654944 10.18632/oncotarget.6510 PMC4823123

[39] Cao Y, Qiu GQ, Wu HQ, Wang ZL, Lin Y, Wu W, et al. Decitabine enhances bortezomib treatment in RPMI 8226 multiple myeloma cells. Mol Med Rep. 2016;14(4):3469–75. 27571872 10.3892/mmr.2016.5658

[40] Huo H, Magro PG, Pietsch EC, Patel BB, Scotto KW. Histone methyltransferase MLL1 regulates MDR1 transcription and chemoresistance. Cancer Res. 2010;70(21):8726–35. 20861184 10.1158/0008-5472.CAN-10-0755 PMC5675022

[41] Yang Y, Zhang J, Wu T, Xu X, Cao G, Li H, et al. Histone deacetylase 2 regulates the doxorubicin (Dox) resistance of hepatocarcinoma cells and transcription of ABCB1. Life Sci. 2019;216:200–6. 30465789 10.1016/j.lfs.2018.11.043

