# Supplementary material for: Promoter hypomethylation drives ABCB1-mediated carfilzomib resistance in multiple myeloma
Source: Clin Epigenetics. 2026 Apr 1;18:59. doi: 10.1186/s13148-026-02115-y (PMC13063960; doi:10.1186/s13148-026-02115-y)
Supplement: Supplementary file 2 — Additional file 2 (.docx). Supplementary Information. Table S1 (clinical annotation for the independent MM subcohort, n = 13); Figure S1 (scatter plot of ABCB1 promoter methylation vs. expression in the independent MM subcohort); Figure S2 (DNMTi treatment results in ARH77WT cells); and Supplementary Methods. [file 13148_2026_2115_MOESM3_ESM.docx]

**Supplementary Information**

**Supplementary Table**

| **ID** | **Age** | **Subtype** | **Bone marrow infiltration** | **Disease status** | **Line of therapy (n)** | | **BTZ** | **CFZ** | ***ABCB1* Promoter methylation** | ***ABCB1* Expression (log_2_[TPM+1])** |
| --- | --- | --- | --- | --- | --- | --- | --- | --- | --- | --- |
| MM1 | 73 | IgG Kappa | 35% | RRMM | | 3 | Yes | No | 1.00% | 0.77 |
| MM2 | 51 | IgG Kappa | 50% | RRMM | | 5 | Yes | No | 5.40% | 0.30 |
| MM3 | 64 | LC Kappa | 90% | RRMM | | 8 | Yes | Yes | 0.90% | 2.16 |
| MM4 | 37 | LC Lambda | 95% | RRMM | | 5 | Yes | Yes | 34.80% | 0.47 |
| MM5 | 59 | IgG Kappa | 75% | RRMM | | 1 | Yes | No | 0.70% | 5.56 |
| MM6 | 64 | IgG Lambda | 50% | NDMM | | 0 | No | No | 14.30% | 0.47 |
| MM7 | 57 | IgA Kappa | 15% | RRMM | | 5 | Yes | No | 1.10% | 0.77 |
| MM8 | 80 | IgG Kappa | 15% | RRMM | | 1 | No | No | 2.60% | 1.25 |
| MM9 | 59 | IgG Kappa | 60% | RRMM | | 5 | No | Yes | 3.00% | 0.50 |
| MM10 | 67 | IgG Kappa | 20% | NDMM | | 0 | No | No | 10.90% | 1.13 |
| MM11 | 68 | IgG Lambda | 35% | RRMM | | 2 | Yes | No | 8.70% | 0.31 |
| MM12 | 69 | IgA Kappa | 90% | RRMM | | 2 | Yes | No | 1.00% | 0.08 |
| MM13 | 62 | LC Kappa | 95% | RRMM | | 7 | Yes | Yes | 0.30% | 7.49 |

Table S1: Clinical annotation for the independent MM subcohort (n = 13). Columns: Sample ID (ID); Age at sampling (Age); MM Subtype; Bone marrow infiltration (%); Disease status (NDMM, RRMM); Line of therapy (n); prior therapy exposures (Bortezomib (BTZ), Carfilzomib (CFZ), Yes = exposure, No = no exposure); *ABCB1* promoter methylation (mean, %); *ABCB1* expression (log_2_[TPM+1]). Abbreviations: NDMM = newly diagnosed multiple myeloma; RRMM = relapsed/refractory multiple myeloma.

**Supplementary Figures**


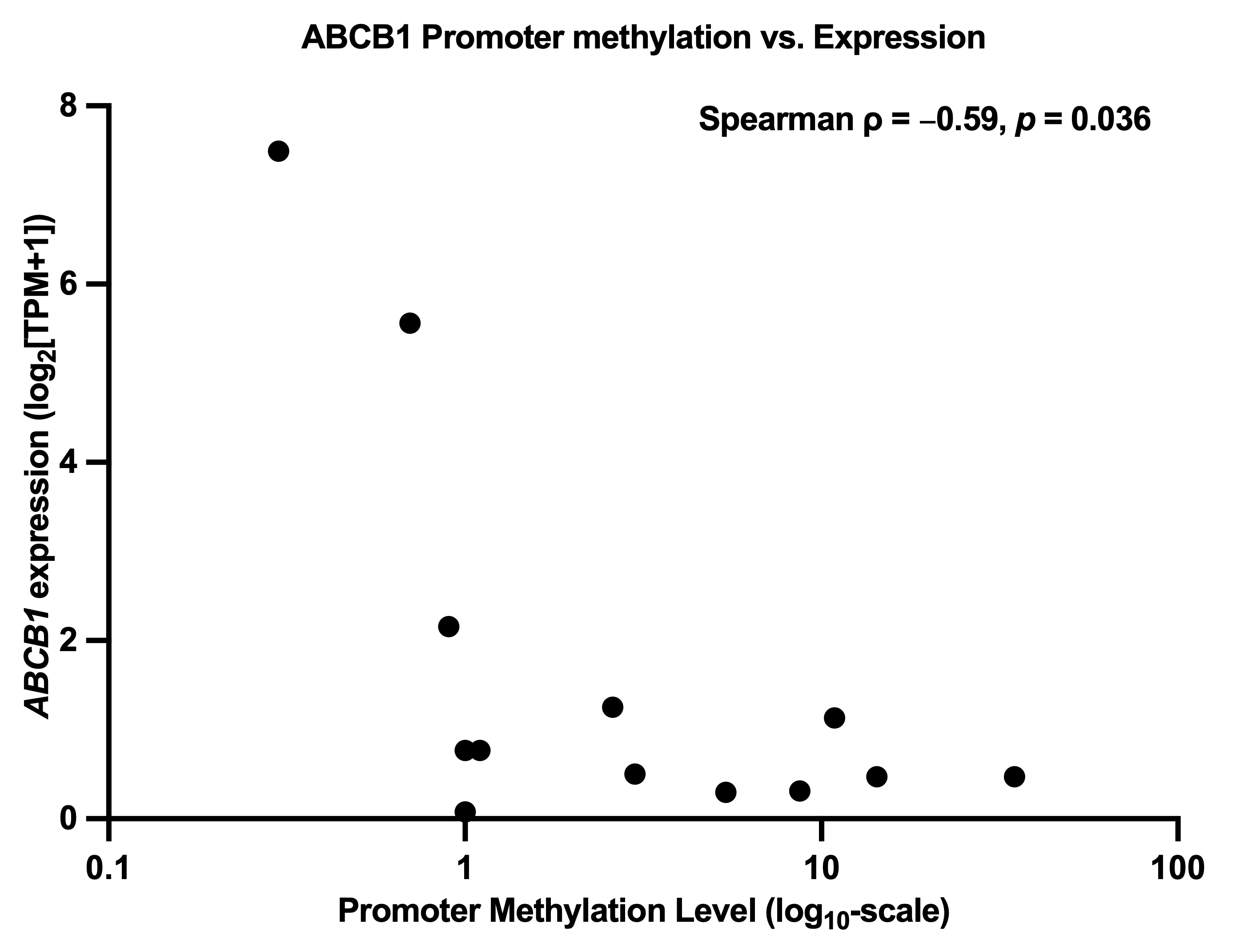


Figure S1: *ABCB1* promoter methylation and gene expression in an independent MM subcohort (n = 13). Scatter plot of the *ABCB1* promoter methylation (%) on a log_10_-scale vs. *ABCB1* expression (log_2_[TPM+1]). An inverse association was observed (Spearman ρ = −0.59, *p* = 0.036). Each dot represents one patient sample.


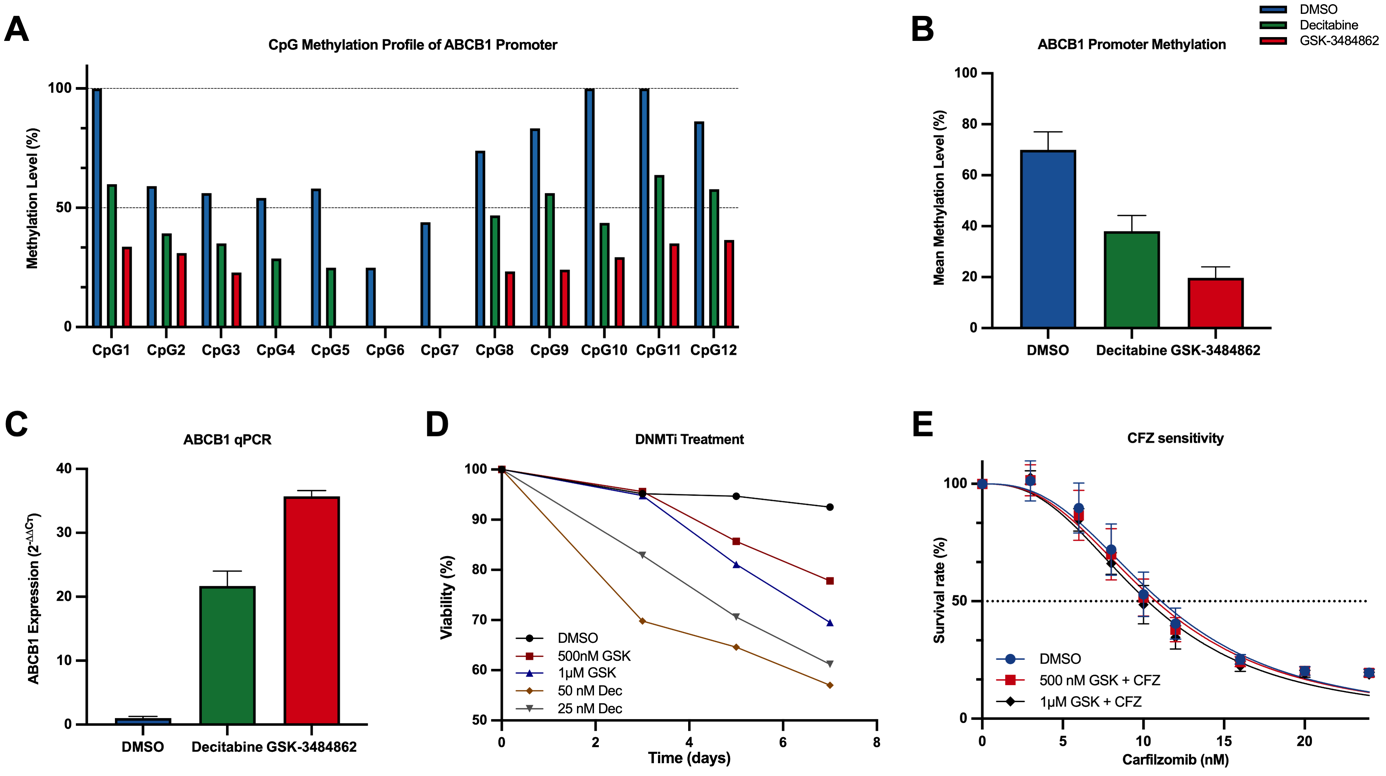


Figure S2: DNMTi treatments of MM cell line. A) Bar plot showing site-specific CpG methylation levels across the ABCB1 promoter region in ARH77WT cells treated with DMSO (blue), 25 nM decitabine (green), or 1 μM GSK-3484862 (red) for seven days. B) Bar plot comparing overall ABCB1 promoter methylation levels between treatment groups. C) Bar plot displaying corresponding ABCB1 expression changes (2^(-ΔΔCT)^) in ARH77WT cells following DNMTi treatments. D) Viability (%) of ARH77WT cells treated with DMSO, GSK (500 nM, 1 µM) or Dec (25 nM, 50 nM), measured at days 0, 3, 5, and 7. E) CFZ sensitivity test (72 h, alamarBlue) co-treated with DMSO or GSK (500 nM, 1 µM).

**Supplementary Methods**

**Whole genome sequencing**

From bone marrow samples of a patient before treatment with carfilzomib and at the time of resistance to carfilzomib, WGS libraries were prepared from 1 μg of DNA with the TruSeq PCR-free library prep kit and 2 × 150 bp paired-end sequences were generated on Illumina NovaSeq 6000 with a median coverage of 99× (CFZ-sensitive sample) and 81× (CFZ-resistant sample). Reads were aligned to the human reference genome (GRCh37, Ensembl annotation) using Isaac aligner (v.03.16.02.19) through the BaseSpace WGS app v.5 (Illumina) with default parameters. A tumor/unmatched normal workflow was used for variant calling. Single nucleotide variants (SNVs) and small indels (<50 bp) were called with Strelka (v.2.4.7) and large-scale structural variants (SVs) with Manta (v.0.28.0). Each variant with a PASS flag was queried against the gnomAD database (v.2.1.1) and variants with global population frequencies >0.05% were excluded to reduce germline calls. Further analysis was performed on protein-altering and splice-site variants only. Copy number variations (CNVs) were called using GATK (v.4.0.8.1, Broad Institute).

**RNA Sequencing**

Total RNA was extracted using AllPrep DNA/RNA Kit (Qiagen). RNA integrity was assessed using the Bioanalyzer 2100 system (Agilent Technologies). Messenger RNA was purified using poly-T oligo-attached magnetic beads, fragmented, and used for cDNA synthesis with random hexamer primers. Libraries were prepared after end repair, A-tailing, adapter ligation, size selection, and amplification using the Novogene NGS RNA Library Prep Set (PT042). Libraries were quality-checked using Qubit and Bioanalyzer before sequencing on an Illumina NovaSeq 6000 platform (Novogene GmbH). Raw reads were quality-filtered using FastQC and Trimmomatic. Clean reads were aligned to the human reference genome (hg38) using HISAT2. Gene expression was quantified with featureCounts and normalized to FPKM values. Differential expression analysis was performed using DESeq2 with adjusted p-value < 0.05 and |log_2_FoldChange| > 1 as significance thresholds. Pathway analyses were conducted using clusterProfiler in R (v.4.1.0).

**Whole-Genome Bisulfite sequencing and data analysis**

***Cell line Samples***

Whole-Genome Bisulfite Sequencing (WGBS) libraries were generated from genomic DNA isolated from each cell line. DNA was fragmented by sonication to 100–300 bp, followed by end-blunting, dA addition at the 3' end, and adapter ligation. Adapter-ligated molecules of 200–300 bp were isolated by agarose gel electrophoresis and subjected to sodium bisulfite conversion using the EZ DNA Methylation-Gold Kit (Zymo Research Corporation). PCR-enriched libraries were purified and sequenced on an Illumina NovaSeq 6000 platform using paired-end 150 bp configuration (CD Genomics Inc.).

***Patient Samples***

For patient samples, genomic DNA spiked with lambda DNA was fragmented to 200–400 bp. Fragmented DNA underwent bisulfite treatment and following methylation sequencing adapter ligation and double-strand DNA synthesis, libraries underwent size selection and PCR amplification. The 200–400 bp insert bisulfite-treated DNA libraries were prepared using the Accel-NGS Methyl-Seq DNA Library Kit for Illumina (Cat No. 30096) and sequenced on an Illumina NovaSeq 6000 platform (Novogene GmbH).

***Bioinformatic Analysis***

For all samples, raw read quality was assessed using FastQC, followed by adapter and low-quality sequence removal with Trim Galore (v.0.6.7). Bisulfite-treated reads were aligned to the human reference genome (hg38) using Bismark (v.0.24.0) with HISAT2 (v.2.2.1) in end-to-end mode. PCR duplicates and redundant portions of paired-end sequences were removed. Methylation levels at individual cytosines were calculated as the ratio of methylated CpG cytosines to total sequenced CpG cytosines [1]. Differentially methylated regions (DMRs) were identified using DSS (Dispersion Shrinkage for Sequencing Data) with a threshold of absolute methylation difference >25% and adjusted p-value <0.05. The GeneHancer database was used to identify the genomic positions of the three *ABCB1* promoter/enhancer regions with the highest "total score" [2]. Methylation data visualization and additional analyses were performed using R (v.4.1.0) with the methylKit and genomation packages.

1. Krueger, F. and S.R. Andrews, *Bismark: a flexible aligner and methylation caller for Bisulfite-Seq applications.* Bioinformatics, 2011. **27**(11): p. 1571-1572.

2. Fishilevich, S., et al., *GeneHancer: genome-wide integration of enhancers and target genes in GeneCards.* Database, 2017. **2017**.
