# Supplementary figures and images for: Promoter hypomethylation drives ABCB1-mediated carfilzomib resistance in multiple myeloma
Source: Clin Epigenetics. 2026 Apr 1;18:59. doi: 10.1186/s13148-026-02115-y (PMC13063960; doi:10.1186/s13148-026-02115-y)

Figure 4C

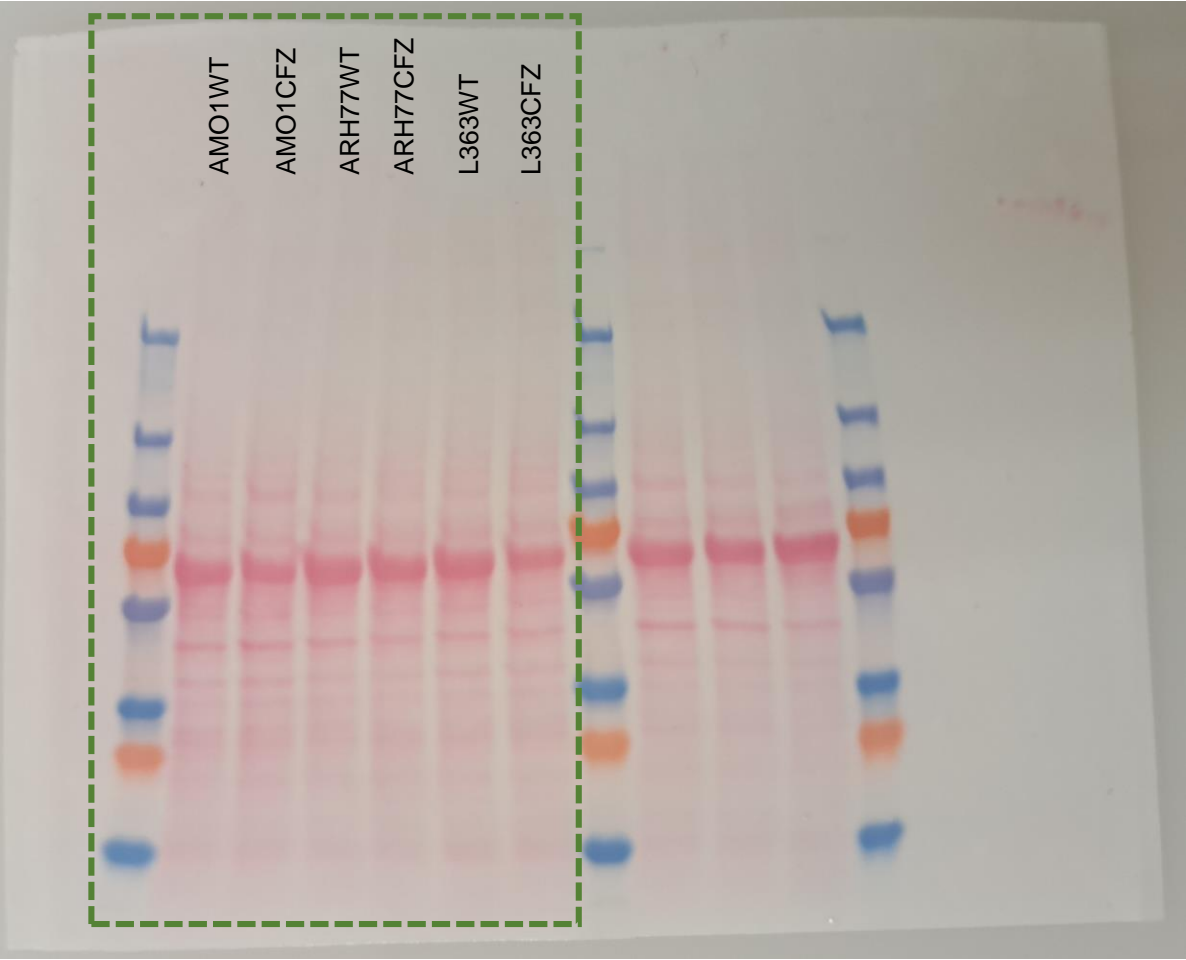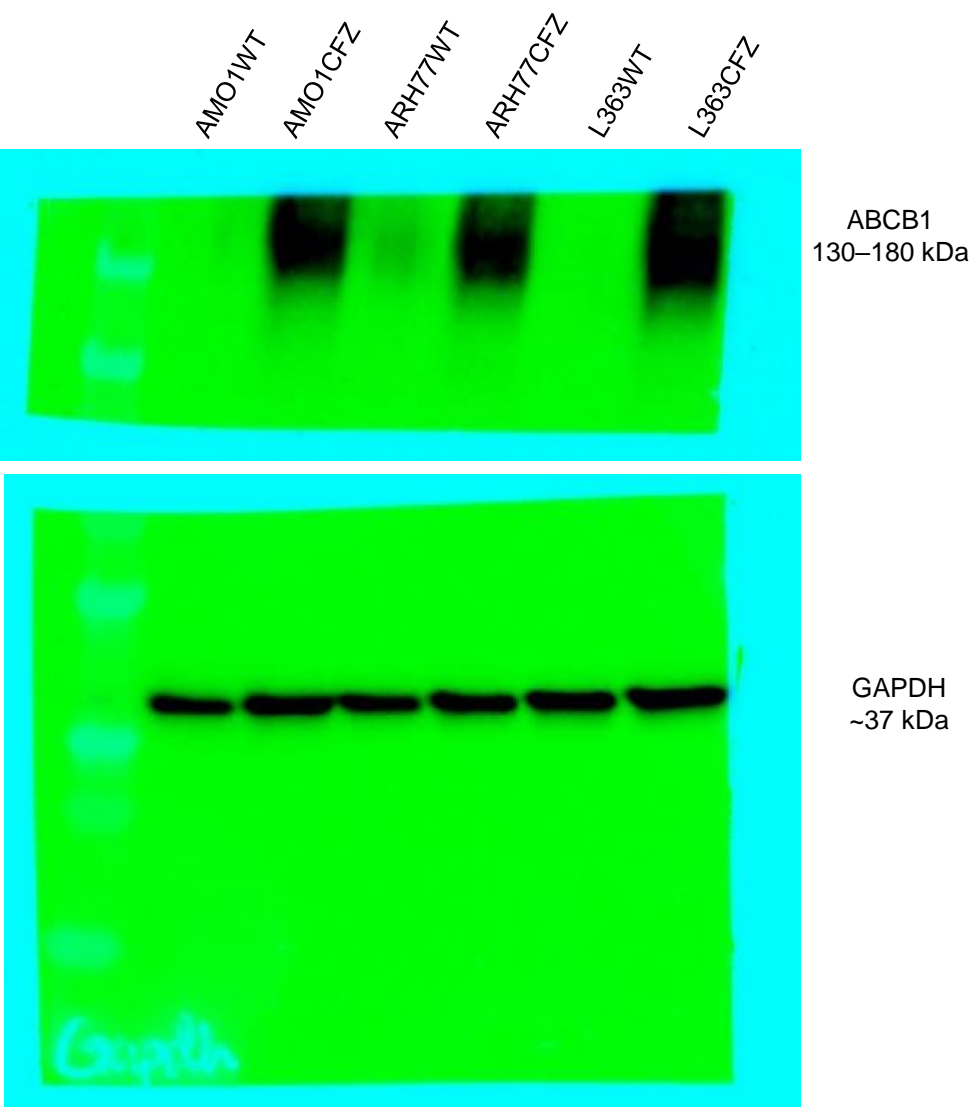

Supplement: Supplementary file 3 — Additional file 3 (.pdf). Uncropped Western blot images for Figure 4C showing ABCB1 and GAPDH protein expression in WT and CFZ-resistant MM cell lines. [file 13148_2026_2115_MOESM2_ESM.pdf]
